# Retraction: Two Birds with One Stone? Possible Dual-Targeting H1N1 Inhibitors from Traditional Chinese Medicine

**DOI:** 10.1371/journal.pcbi.1014360

**Published:** 2026-06-09

**Authors:** The PLOS Computational Biology Editors

The *PLOS Computational Biology* Editors retract this article [1] due to concerns about compromised peer review. We regret that the issues were not addressed prior to the article’s publication.

All authors either did not respond directly or could not be reached.
